# Testing a Novel Wearable Device for Motor Recovery of the Elbow Extensor Triceps Brachii in Chronic Spinal Cord Injury

**DOI:** 10.1523/ENEURO.0077-23.2023

**Published:** 2023-07-26

**Authors:** Maria Germann, Stuart N. Baker

**Affiliations:** Institute of Biosciences, Faculty of Medical Sciences, Newcastle University, Newcastle upon Tyne NE2 4HH, United Kingdom

**Keywords:** electrical stimulation, plasticity, reticulospinal, spinal cord injury

## Abstract

After corticospinal tract damage, reticulospinal connections to motoneurons strengthen preferentially to flexor muscles. This could contribute to the disproportionately poor recovery of extensors often seen after spinal cord injury (SCI) and stroke. In this study, we paired electrical stimulation over the triceps muscle with auditory clicks, using a wearable device to deliver stimuli over a prolonged period of time. Healthy human volunteers wore the stimulation device for ∼6 h and a variety of electrophysiological assessments were used to measure changes in triceps motor output. In contrast to previous results in the biceps muscle, paired stimulation: (1) did not increase the StartReact effect; (2) did not decrease the suppression of responses to transcranial magnetic brain stimulation (TMS) following a loud sound; (3) did not enhance muscle responses elicited by a TMS coil oriented to induce anterior-posterior current. In a second study, chronic cervical SCI survivors wore the stimulation device for ∼4 h every day for four weeks; this was compared with a four-week period without wearing the device. Functional and electrophysiological assessments were repeated at week 0, week 4, and week 8. No significant changes were observed in electrophysiological assessments after paired stimulation. Functional measurements such as maximal force and variability and speed of trajectories made during a planar reaching task also remained unchanged. Our results suggest that the triceps muscle shows less potential for plasticity than biceps; pairing clicks with muscle stimulation does not seem beneficial in enhancing triceps recovery after SCI.

## Significance Statement

A wearable device which pairs auditory clicks with electrical stimulation over a muscle was previously shown to strengthen putative reticulospinal connections to the biceps brachii muscle. Here, we tested whether this device could also strengthen connections to the triceps muscle, both in healthy volunteers and in people with spinal cord injury (SCI). Surprisingly, there was no evidence of an effect. This suggests that connections to triceps differ from those to biceps in their ability to undergo plastic changes. This may underlie the poor recovery of triceps compared with biceps typically seen after spinal cord injury.

## Introduction

Damage to the corticospinal tract through spinal cord injury (SCI) often results in decreased voluntary control of muscles below the level of injury, or even full paralysis. The recovery of any remaining function following SCI is associated with plastic changes and reorganization of neural pathways.

While extensive axonal regeneration in the CNS is absent, even the most severe SCIs often spare regions of white matter (Kakulas, 1999; Petersen et al., 2012; Angeli et al., 2014; Barthelemy et al., 2015) and surviving CST fibers in these bridges may contribute to recovery, for example, through spontaneous sprouting (Rosenzweig et al., 2010).

Alternatively, other descending pathways may provide a route for motor command transmission. Monkeys show a remarkable recovery after CST lesions (Lawrence and Kuypers, 1968a, b) and regain most of their gross locomotor functions, with motor outputs from the red nucleus strengthening (Belhaj-Saïf and Cheney, 2000) to provide a substrate for recovery. However, the rubrospinal tract may be weak or nonexistent in humans (Nathan and Smith, 1955; Onodera and Hicks, 2010), which could explain the stark difference in functional recovery after CST damage.

In the absence of a rubrospinal tract, humans may have to rely on medial brainstem pathways, including the reticulospinal tract (RST), after damage to the CST. While the CST has become the dominant pathway in primates, reticulospinal connections to motoneurons strengthen after CST lesions, partially restoring lost synaptic drive (Zaaimi et al., 2012, 2018). Increased inputs from both CST and RST could help recovery after SCI. Importantly, reticulospinal neurons may show more potential for plasticity than corticospinal neurons after injury (Vavrek et al., 2007; Zörner et al., 2014).

Noninvasive methods to modulate the RST in humans are limited, but exploiting the fact that reticulospinal neurons receive extensive afferent input (Leiras et al., 2010) and auditory stimuli excite reticular neurons (Irvine and Jackson, 1983; Fisher et al., 2012), we previously built a portable device capable of delivering stimuli to the motor point of a muscle paired with an auditory click, which is able to induce plastic changes in motor output (Foysal et al., 2016; Germann and Baker, 2021). Using this device at home for four weeks produced significant improvements in upper limb function for stroke survivors (Choudhury et al., 2020), which were retained for at least four weeks after device stimulation ceased.

Elbow extensors show notoriously limited recovery compared with elbow flexor muscles in individuals with cervical SCI (Ditunno et al., 1992, 2000; Calancie et al., 2004; McKay et al., 2011). This asymmetrical recovery of function is also commonly seen after stroke, with survivors often exhibiting flexor spasm and extensor weakness (Twitchell, 1951; Cauraugh et al., 2000; Kamper et al., 2003). Most cervical spinal cord injuries affect more than one spinal segment (Schaefer et al., 1989; Benavides et al., 2020), making it unlikely that CST projections to elbow extensors would be regularly more damaged compared with elbow flexors. The location of motoneurons that innervate these muscles also largely overlap (Schirmer et al., 2011).

The imbalanced recovery might be because of the differential control of flexor and extensor motoneurons by corticospinal and reticulospinal pathways (Sangari and Perez, 2020). Indeed, after CST lesion in monkeys, reticulospinal connections strengthen to forearm flexor and intrinsic hand muscles, but not to forearm extensors (Zaaimi et al., 2012). In the healthy state, the RST has been shown to preferentially facilitate ipsilateral flexors and contralateral extensors (Davidson and Buford, 2006).

Our previous work using the wearable device to modulate motor output (Foysal et al., 2016), most likely by inducing subcortical plasticity (Germann and Baker, 2021), targeted the elbow flexor biceps brachii. The same protocol was able to improve upper limb function after stroke (Choudhury et al., 2020) by targeting forearm extensor muscles. Here, we investigated whether this protocol could also generate plastic changes in triceps. In both healthy individuals and chronic cervical spinal cord injury survivors, we found no significant changes in electrophysiological measurements from triceps, or in functional measures of elbow extension. This suggests that triceps may be more resistant to stimulation protocols which induce plasticity in subcortical pathways.

## Materials and Methods

### Single-day study in healthy volunteers: subjects

In total, 11 healthy volunteers (six females and five males; 18–35 years old, right-handed by self-report) participated in the study. Four subjects took part in two experiments. If participants took part in several stimulation paradigms, each session was separated by at least 7 d. All subjects gave written informed consent to the experimental procedures, which was approved by the appropriate ethics committee. The study was performed in accordance with the guidelines established in the Declaration of Helsinki, except that the study was not preregistered in a database.

### Single-day study in healthy volunteers: experimental paradigm

The single-day study in healthy subjects followed the same paradigm as our previous work targeting the biceps muscle (Germann and Baker, 2021).This required subjects to come to the laboratory at around 9 A.M., where baseline assessments were conducted. They were then fitted with a wearable electronic device to deliver paired electrical and auditory stimuli, and the subject left and continued their normal daily activities. In the evening, the subject returned, the device was removed, and the assessments repeated. The order of the various assessments was randomized for a given subject, but kept the same for the morning and evening recordings in that person. The two assessment sessions were at least 6 h apart.

For this study, conducted over 1 d, the following assessments were used at baseline and after wearing the device: StartReact, loud sound with MEP, MEPs with different coil orientations (see below, sections beginning ‘Assessment’). Assessments used in this study are summarized in Table 1.

**Table 1:** Summary of assessments used in the two separate studies

Assessments	Single-day study in healthy volunteers	SCI study
StartReact	x	x
Loud sound with MEP	x	x
MEPs with different coil Orientation	x	x
Force		x
Planar reaching task		x
CUE Questionnaire		x

Seven subjects wore the earpiece in the contralateral ear and 8 subjects wore the earpiece in the ipsilateral ear (relative to the stimulated right triceps).

### SCI study: subjects

Subjects needed to fit the following inclusion criteria to be eligible for the study: at least 18 years of age; chronic (more than one year) cervical (C2–C7) injury; detectable electromyography (EMG) activity in triceps; without upper limb fracture or subluxation/dislocation of joints within last six months; be able to follow study instructions; willing to provide written informed consent (if necessary, through an independent witness, should the subject be unable to write themselves because of their injury).

### SCI study: trial design

This study was designed as an interventional randomized crossover trial. The trial was registered in the ISRCTN registry (ISRCTN15025040). After completing the baseline session (week 0) which included all assessments (see below, sections begining ‘Assessment’; Table 1), participants were randomized to one of two groups using custom MATLAB code. Group A was issued the device to be used at home for four weeks, returned for a second assessment session (week 4) and then stopped wearing the device and returned in another four weeks (week 8). Group B first did not wear the device and returned for a second session (week 4), when they were issued the device to use for the remaining four weeks (week 8). For the period spent using the device, participants were instructed to wear it for at least 4 h (total usage) every day, in line with the previous study in stroke survivors (Choudhury et al., 2020). Because of the nature of the intervention and control (device vs no device), subjects and researchers were not blinded.

The device can be worn during all activities with the exception of showering and sleeping. Participants were carefully shown how to place the electrodes and given a detailed information pamphlet on how to use the device, as well as sufficient electrodes to last them for the month. Participants chose whether they wanted to stimulate the left or right triceps for the duration of the study (11 chose right arm, 4 chose left arm). Participants were free to choose the stimulated side, to maximize the benefit to them from any potential improvement.

### SCI study: adverse events

An adverse event (AE) was defined as any untoward medical event, including abnormal laboratory values, occurring during the use of the study device, but not necessarily causally related to it. Events with onset before start of study device use were not considered as adverse events unless there was an increase in severity and/or frequency following use of the study device. No AEs were reported during the study.

### Electromyography (EMG) recordings

EMG was recorded through surface electrodes (disposable Kendall H59P Electrodes, Covidien) secured on the skin over the belly of the triceps, biceps, and deltoid muscle.

For the 1-d study in healthy subjects, the electrodes over the right triceps muscle were used for both EMG recording and for wearable stimulation, so they were kept in place for the whole day, ensuring consistency between the morning and evening sessions. For the SCI study, electrodes had to be repositioned for each session.

EMG signals were amplified and filtered (30–2000 Hz, gain 1000) with a bioamplifier (D360 Amplifier, Digitimer), digitized at a sampling rate of 5 kHz (CED Micro 1401 with Spike2 software, Cambridge Electronic Design) and stored on a computer for off-line analysis. Where necessary for a given assessment (see below, sections beginning ‘Assessment’), the level of triceps EMG activity was displayed continuously on a computer screen via series of colored bars, allowing subjects to maintain a constant isometric contraction. Physiologic measurements were acquired at 10–20% of MVC across conditions. The exact level of MVC varied across participants, but was kept constant for all assessments of each participant.

### Transcranial magnetic stimulation (TMS)

Transcranial magnetic stimuli were applied using a figure-of-eight coil through a Magstim 200^2^ magnetic stimulator (Magstim) with a monophasic current waveform. We determined the optimal position for eliciting a motor evoked potential (MEP) in the triceps muscle (hotspot) by moving the coil tangential to the scalp, at an angle of 45° to the midline, with the handle pointing laterally and posteriorly, in small steps along the arm representation of M1. The hotspot was defined as the region where the largest MEP in the triceps muscle could be evoked (Rothwell et al., 1999). Unless stated otherwise, the magnetic coil was held in this orientation over the left hemisphere for subsequent measurements, to induce currents in the brain that flowed perpendicular to the presumed line of the central sulcus in a posterior-anterior (PA) direction.

Active motor threshold (AMT) was defined as the stimulator intensity sufficient to elicit a visible MEP in at least five out of 10 consecutive stimuli, in the triceps contralateral to the stimulus with an active contraction of 10–20% maximal voluntary contraction (MVC).

A Polaris Vicra camera (Northern Digital Inc.) tracked both coil and head position, allowing the site of stimulation to be marked using the Brainsight neuronavigation system (Rogue Research Inc.). This ensured a stable coil location throughout the experiment, and allowed us to return to the same site in all sessions.

### Assessment: StartReact

StartReact was examined using a previously reported paradigm (Baker and Perez, 2017; Germann and Baker, 2021), which measures reaction time in an EMG recording in response to a visual cue [visual reaction time (VRT)], a visual plus quiet auditory cue [visual-auditory reaction time (VART)], and a visual plus loud auditory cue [visual-startle reaction time (VSRT)]. The acceleration of reaction time between VART and VSRT reflects the action of the RST (Valls-Solé et al., 1999; Carlsen et al., 2004; Rothwell, 2006; Baker and Perez, 2017; Smith et al., 2019; Tapia et al., 2022). Some previous studies on StartReact have measured startle responses from the sternocleidomastoid (SCM) muscle, and excluded reaction times measured when this muscle was not activated (Valls-Solé et al., 1999; Carlsen et al., 2004; Honeycutt et al., 2013). We and others have previously found that SCM activity is unreliable (Honeycutt et al., 2013; Baker and Perez, 2017; Dean and Baker, 2017), and can be generated both in response to startling and nonstartling cues. In this study, we therefore simply included all reaction times measured from the relevant cue, as in our previous publications (Baker and Perez, 2017; Dean and Baker, 2017; Choudhury et al., 2019; Germann and Baker, 2021).

Subjects were seated with the arm flexed at the elbow by 90° and secured in an arm restraint.

A green light-emitting diode (LED) was located ∼1 m in front of the subject. Subjects were instructed to push against the restraint (elbow extension) as quickly as possible after the LED illuminated. EMG was recorded from both the triceps and biceps muscles, and reaction time measured as the time from cue to onset of the EMG burst. Three types of trial were randomly interleaved (20 repeats per condition; intertrial interval 5–6 s): LED illumination alone (VRT), LED paired with a quiet sound (80 dB, 500 Hz, 50 ms, VART), LED paired with a loud sound (500 Hz, 50 ms, 120 dB, VSRT). Subjects were initially presented with five consecutive loud sounds, without performing the task, to allow familiarization and to habituate the overt startle reflex.

Data were analyzed trial-by-trial using a custom MATLAB program which identified the reaction time as the point where the rectified EMG exceeded the mean ± 7 SD of the baseline measured 0–200 ms before the stimulus. Every trial was inspected visually, and erroneous activity onset times (caused, for example, by electrical noise artefacts) were manually corrected. This yielded a reaction time distribution for each condition.

### Assessment: loud sound with MEP

A paradigm similar to previous studies (Furubayashi et al., 2000; Tazoe and Perez, 2017; Germann and Baker, 2021) was used. Loud (500 Hz, 120 dB SPL) auditory stimuli (LAS) were given 50 ms before a TMS pulse over M1; this elicits a smaller MEP than the same TMS pulse given alone, which is believed to reflect cortical inhibitory processes. The interval used was selected as it gave the largest suppression in a previous detailed examination of these effects (Furubayashi et al., 2000). Sounds were given through two audio speakers located on a table ∼1 m in front of the subject. The sound ended at the time of the test stimulus. To avoid stimulus predictability, the intertrial interval was chosen randomly between 20.5 and 23 s (uniform distribution). At the beginning of the study, five consecutive loud sounds were presented to habituate the startle reflex. During testing subjects were seated with the arm flexed at the elbow by 90° and pushed against the restraint at 10–20% of MVC. The level of triceps EMG activity was displayed continuously on a computer screen via a series of colored bars, allowing subjects to maintain a constant isometric contraction, which was kept the same across sessions for that subject. The test stimulus intensity was adjusted to be 130% of the AMT. Ten test MEPs and 10 conditioned MEPs were measured in each condition, in randomized order.

### Assessment: MEPs with different coil orientations

By holding a figure-of-eight TMS coil in different orientations, activation can be biased toward different indirect (I) waves (Sakai et al., 1997; Ziemann and Rothwell, 2000). With the coil tangential to the scalp, at an angle of 45° to the midline with the handle pointing laterally and posteriorly, a posterior-anterior (PA) current is induced in the brain, which mainly recruits early I waves. By contrast, in the reverse orientation [handle pointing medial and anterior; anterior-posterior (AP)-induced current], predominantly late I waves are recruited. Here, we measured MEPs with different coil orientations to compare changes in direct versus indirect corticospinal pathways.

MEPs in the triceps muscle were measured from the contralateral hemisphere in the two different coil orientations. As for the measurements of MEPs described above, the figure-of-eight coil was first held to induce a posterior-anterior (PA) current in the brain. The optimal scalp position was located with this orientation, and marked in the Brainsight neuronavigation software. This site was then used for stimulation to induce an anterior-posterior (AP) current in the brain, by rotating the coil so that the handle faced anterior and medial, as the current direction does not significantly influence the position of the hotspot (Sakai et al., 1997; Arai et al., 2005). Within each subject, we randomized the order of coil orientations (PA vs AP); the same order was used for the baseline and all following assessments. Stimulus intensity was set to 1.1× active motor threshold (two separate thresholds were measured, one for each coil orientation). Subjects were seated with the arm flexed at the elbow by 90° and secured in a restraint.

The level of triceps EMG activity was displayed continuously on a computer screen via a series of colored bars. Twenty MEPs were recorded (5-s interstimulus interval) while subjects maintained a constant isometric contraction of 10−20% MVC by pushing against the arm restraint.

### Assessment: MVC and force

Subjects were asked to perform three brief (3 s) maximal contractions by pushing or pulling against the arm restraint, for elbow extension or elbow flexion respectively. Each contraction was separated by 60 s. Force was measured with a dynamometer built into the arm restraint.

### Assessment: planar reaching task

**Figure 1.**
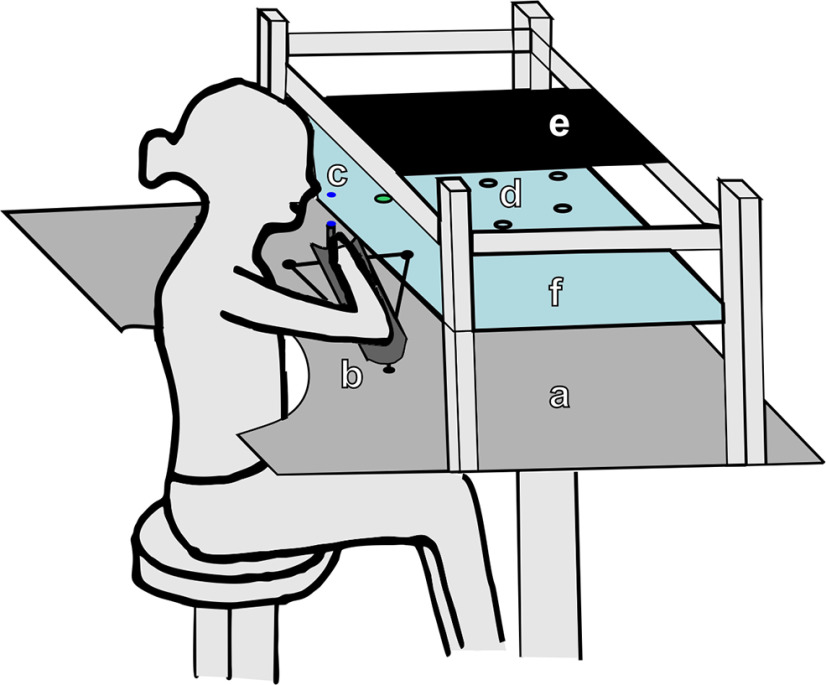
Schematic representation of the planar reaching task. ***a***, Smooth table surface. ***b***, Arm support air sled. ***c***, LED cursor, positioned on top of the air sled. ***d***, Targets, projected to plane of the arm from ***e***, LED lights panel via ***f***, two-way mirror.

Participants sat at a custom-built table (Fig. 1) with their trunk secured to a high-backed chair. Table height was adjusted to bring shoulder, elbow, and wrist close to the same horizontal plane. The reaching arm was supported on an air cushion sled that used continuous pressurized airflow to support the limb against gravity and allowed near frictionless movements. Participants were instructed to make 20 straight movements to visual targets (160 trials in total), projected using a two-way mirror to appear in the same plane as a blue LED cursor (diameter 3 mm) attached to the sled.

The targets consisted of a start position at the center and 8 targets of equal angular range around the central target, each with 1-cm radius. The targets were arranged at a distance 10 cm radially from the center start position. One target at a time would light up green. The target turned red as soon as the cursor reached the target area. Participants had to hold the cursor inside the target for 800–1209.6 ms, before the center target would light up green and participants returned to start position. Targets appeared in a pseudo-randomized order.

Arm position was tracked using a Polhemus Liberty motion tracking system (Polhemus) with a sensor built into the support sled.

Despite the arm support, two participants were unable to perform the required reaching movement and analysis is based on the remaining 13 participants.

Targets for participants who chose to use their left arm for the study were mirrored across the midline, so comparable muscle groups were used for each target across subjects.

Trajectories of SCI participants were compared with data from 18 healthy volunteers performing the same task with their right arm (13 females and 5 males; 18–35 years old, right-handed by self-report).

### Assessment: CUE questionnaire

The Capabilities of Upper Extremity (CUE) Questionnaire measures upper extremity functional limitations in individuals with tetraplegia (Marino et al., 1998). It is a 32-item questionnaire evaluating perceived difficulty completing actions using the right (15 items), left (15 items), or both (two items) upper extremities. The participant gave each action a score from 0 to 4, with higher scores reflecting less limitation. Left and right arm function can be derived separately. The scores for both arms were summed and compared across sessions.

### Intervention: wearable device

The wearable device was similar to that used in our previous work (Choudhury et al., 2020). It comprised a plastic box containing an electrical stimulator and audio amplifier, powered by an internal battery which could be recharged via a standard USB-C port. The wearable device generated constant-current electrical stimulation to the right biceps muscle through surface electrodes (220-V compliance, 0.15-ms pulse width). A knob on the device allowed adjustment of the stimulus intensity, which was set to be just below the motor threshold (defined as a visible muscle twitch). Auditory stimuli were generated by delivering a 0.1 ms wide, 3.3-V square excitation pulse into a miniature earpiece; this produced a brief click with an intensity of 110 dB SPL.

Based on calculations provided by Rosengren et al. (2010) and Foysal et al. (2016), this intensity corresponds to an A-weighted intensity of 68 dB LA_eq_ when delivered at 0.66 Hz, and 71 dB LA_eq_ when delivered at 1.32 Hz; assuming device usage for at least 8 h. This is well below the recommended safe limit for noise exposure of 85 dB LA_eq_ given by the United Kingdom’s Control of Noise at Work Regulations (The Health and Safety Executive, 2005). The device can therefore potentially be used every day for at least 8 h at these levels without concern.

For the 1-d study in healthy subjects, seven subjects wore the earpiece in the contralateral ear and eight subjects wore the earpiece in the ipsilateral ear (relative to the stimulated right triceps). For the SCI study, the earpiece was always placed contralateral to the stimulated triceps muscle.

Stimuli were delivered with an intertrial interval of 1250–1750 ms (chosen at random from a uniform distribution); the delay between electrical stimulation and auditory click was 10 ms. This interstimulus interval was chosen so that the afferent volley should arrive at the brainstem just before RST cell activation by the click.

The device logged to flash memory how many stimuli were given each time it was switched on, allowing the experimenter to check how often it had been used at the end of the four-week usage period.

### Data analysis

Data were analyzed using custom scripts written in the MATLAB environment (R2017a, MathWorks). Statistical tests were performed using MATLAB and IBM SPSS Statistics for Windows, version 24 (IBM Corp.).

EMG traces were full-wave rectified and then averaged. MEP amplitude was measured as the area under the curve of this average.

Where MEPs were conditioned by loud sound, the amplitude of the conditioned MEP was expressed as a percentage of the unconditioned MEP.

The StartReact effect was measured as the difference between the mean VART and VSRT.

For the assessment which conditioned MEPs with loud sound, a one sample *t* test was performed to compare the normalized conditioned MEP amplitude against 100%, to evaluate the effect of conditioning at baseline.

For each experiment, individual paired *t* tests were used to compare the measurements of each session to the measurements at baseline.

For the SCI study, the session preceding device usage was taken as the “before” (week 0 for group A; week 4 for group B) and the session immediately after wearing the device was taken as the “after” (week 4 for group A; week 8 for group B) for the “device” condition. The same principle was used for the “no device” condition; with the “before” session being week 4 for group A and week 0 for group B and the “after” session being week 8 for group A and week 4 for group B. Differences between before and after sessions were compared with paired *t* tests.

Where necessary, the procedure introduced by Benjamini and Hochberg (1995) to correct for multiple comparisons was used. Cohen’s *d* was used to measure effect size.

To assess the overall movement quality during planar reaching, Mahalanobis distance squared (MDC^2^) was calculated using functional principal component analysis (PCA), a generalization of traditional PCA (Kitago et al., 2015; Goldsmith and Kitago, 2016; Cortes et al., 2017). MDC^2^ was derived from a comparison between reaching trajectories of SCI participants and trajectories from a group of healthy controls (*N* = 18).

For the reaching task, two-way repeated measures ANOVAs were performed with factors time (before, after) and target (Targets 1–8) on speed, distance, and MDC^2^ of trajectories.

Additionally, two-way repeated measures ANOVAs were performed with factors target (Targets 1–8) and condition (device, none) on speed, distance, and MDC^2^ differences (after-before). Speed, distance, and MDC^2^ at baseline (week 0 for both groups A and B) were also assessed with a repeated measures ANOVA with factor target (Targets 1–8).

The significance level was set at *p* < 0.05. Descriptive statistics are presented as mean ± SD.

Binomial tests were performed to determine whether the number of subjects showing a certain change were more than expected by chance based on a binomial distribution.

## Results

### Single-day study in healthy volunteers

**Figure 2.**
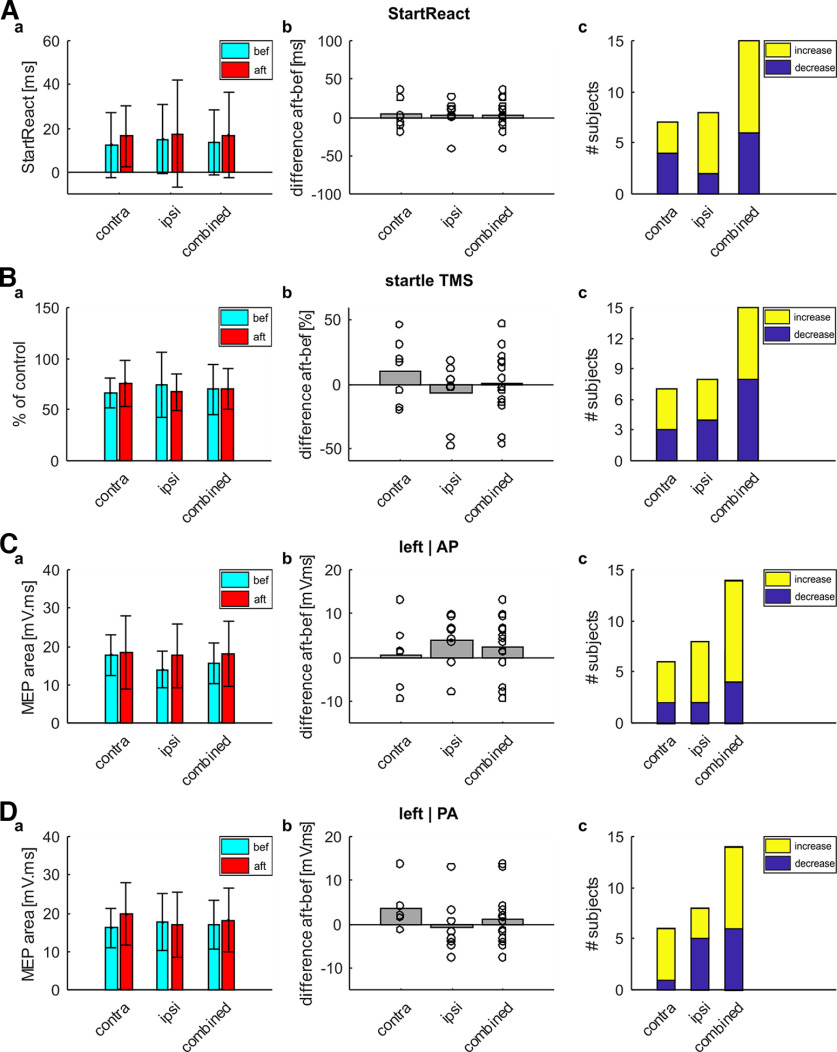
Group results for the single-day study in healthy triceps. ***A***, Results for the StartReact assessment (contralateral audio *N* = 7, ipsilateral audio *N* = 8, both groups combined *N* = 15). The figure represents StartReact effect (difference between VART and VSRT). ***a***, StartReact effect before (cyan) and after (red) wearable device stimulation. Colored bars represent group means; error bars indicate SDs. ***b***, difference in StartReact effect between before and after wearable device stimulation. A positive difference indicates a more pronounced StartReact effect after wearable device stimulation. Bars represent group means; circles show single subject values. ***c***, Number of subjects showing an increase (yellow) or decrease (blue) in StartReact effect after wearable device stimulation. ***B***, Results for the conditioned MEPs with loud sounds. The figure represents conditioned MEP amplitude normalized to unconditioned amplitude (as percentage of control). Layout is the same as for ***A***. ***C***, Results for the coil orientation assessment when stimulation with an AP coil orientation. The figure represents MEP amplitude as area under the curve. For one subject stimulation threshold was too high to detect AP MEPs, so subject was excluded for the coil orientation assessment (contralateral audio *N* = 6, ipsilateral audio *N* = 8, combined *N* = 14). Layout is the same as for ***A***. ***D***, Results for the coil orientation assessment when stimulation with a PA coil orientation. The figure represents MEP amplitude as area under the curve. For one subject stimulation threshold was too high to detect AP MEPs, so subject was excluded for the coil orientation assessment (contralateral audio *N* = 6, ipsilateral audio *N* = 8, combined *N* = 14). Layout is the same as for ***A***.

Figure 2 presents group results for the single-day study in healthy volunteers, for the StartReact (Fig. 2*A*), LAS with TMS (Fig. 2*B*), MEPs elicited with AP coil orientation (Fig. 2*C*) and MEPs elicited with PA coil orientation (Fig. 2*D*) assessments. Seven participants wore the earpiece in the contralateral ear and eight in the ipsilateral ear. Both groups received electrical stimulation to the right triceps. Because of the low number of participants, results were also combined to a total *N* = 15.

**Figure 3.**
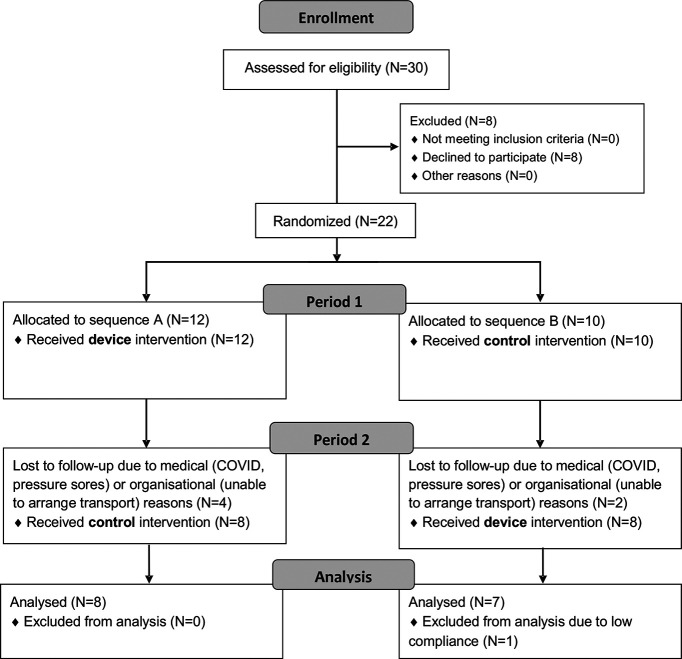
Consort diagram. Trial flow diagram for the SCI study. Participants were randomized into two groups (***A***, ***B***), which both spent four weeks wearing the device and four weeks without wearing the device, but in opposite order. Subsequent analysis is based on the remaining 15 participants.

There was no significant difference in StartReact effect after wearable device stimulation (*p* = 0.628 contralateral, *p* = 0.732 ipsilateral, *p* = 0.538 combined; Fig. 2*Aa*).

Figure 2*B* shows the results when MEPs elicited by TMS were conditioned by a loud sound beginning 50 ms before the magnetic stimulus. Our previous study in the biceps muscle (Germann and Baker, 2021) showed significantly suppressed MEPs at baseline. For the triceps, the group wearing the earpiece contralaterally did show a significant MEP suppression (one sample *t* test *p* < 0.001) at baseline (Fig. 2*Ba*); however, for the group wearing the audio ipsilaterally, this suppression did not quite reach significance (*p* = 0.059). When the groups were combined, MEPs were significantly suppressed at baseline (*p* < 0.001) as expected from the literature (Furubayashi et al., 2000; Tazoe and Perez, 2017). However, there was no change in conditioned MEP suppression after wearable device stimulation (*p* = 0.340 contralateral, *p* = 0.426 ipsilateral, *p* = 0.939 combined; Fig. 1*Ba*).

There was no change in MEP size in the triceps after wearable device stimulation, when the coil was held in the AP (*p* = 0.851 contralateral, *p* = 0.111 ipsilateral, *p* = 0.203 combined; Fig. 2*C*) or PA (*p* = 0.160 contralateral, *p* = 0.790 ipsilateral, *p* = 0.494 combined; Fig. 2*D*) orientation over the left hemisphere (contralateral to the arm stimulated by the wearable device).

Overall, there did not seem to be any changes to responses in the triceps muscle after wearable device stimulation for a single day. These results are in stark contrast to our results in the biceps muscle (Germann and Baker, 2021), which used the same stimulation protocol and duration.

While there was no significant majority (compared with the binomial distribution) of subjects showing a certain direction of change for any of the assessments (Fig. 2*Ac*,*Bc*,*Cc*,*Dc*), most subjects receiving the clicks ipsilaterally showed an increase in StartReact, attenuation of MEP suppression in response to LAS and facilitation of MEPs elicited with AP-induced current, as previously seen in biceps. All of these measures had two clear outlier subjects which could have a disproportionate impact on our results.

### SCI study

For the SCI trial, a total of 22 participants with chronic cervical spinal cord injury were recruited to the study (Table 2). All participants had to complete three sessions, each separated by approximately four weeks. Participant flow is depicted in Figure 3. Six participants dropped out during the course of the study, as they were unable to attend all three session appointments within an acceptable number of days of the target date (>8 d difference). One subject was excluded from group analysis, as their usage of the device was too low during the four weeks (see below, this section). Subsequent analysis is based on the remaining 15 participants (six females, nine males, 45.2 ± 14.0 years old; Table 2).

**Table 2:** SCI participants

Participant no.	Age (years)	Gender	Level	Injury (years)	Arm	Device usage (d)
1	35	F	C5 incomplete	2	R	28
2	32	F	C5–C6 incomplete	3	R	27
3	52	M	C3–C4 incomplete	6	R	28
4	67	M	C3–C4 incomplete	13	R	28
5	39	M	C5–C6 incomplete	17	R	28
6	62	M	C5–C6 complete	28	R	28
7	45	F	C4 incomplete	17	R	28
8	30	M	C4 complete	11	R	31
9	34	M	C4 complete	5	R	29
10	67	F	C3–C7 incomplete	4	R	28
11	60	F	C2–T2 incomplete	15	L	35
12	22	M	C5 incomplete	5	L	28
13	47	F	C4–C5 incomplete	3	L	28
14	39	M	C4–C5 incomplete	2	R	28
15	47	M	C6 complete	22	L	28
16	34	M	C4–C5 incomplete	10	R	40

Participant number 16 was subsequently excluded from analysis because of low compliance.

Of these 15 participants, one subject had to be excluded from the StartReact Assessment because of equipment failure during one session, two subjects were unable to perform the required reaching movements for the planar reaching task and had to be excluded for that task and in two subjects it was not possible to elicit MEPs with the AP coil orientation, so these individuals had to be excluded for the coil orientation assessment.

**Figure 4.**
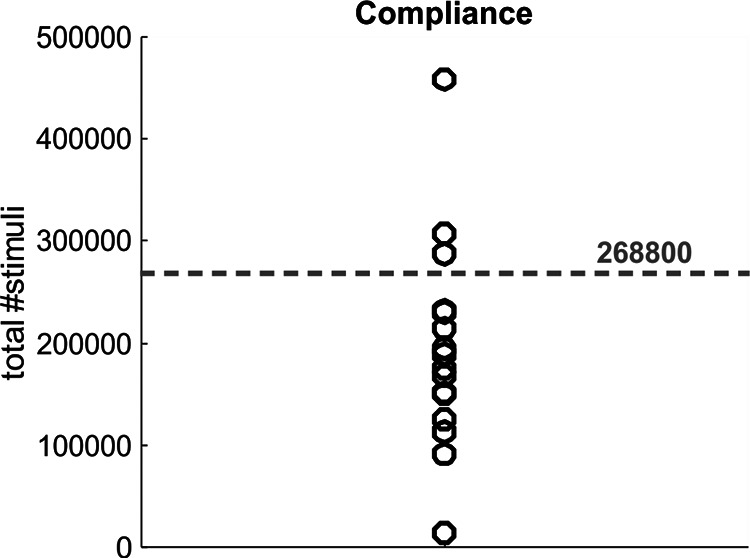
Compliance of device usage in SCI participants. The device recorded the number of stimuli given over the four-week period. Gray dashed line represents total number of stimuli received if the device was used as instructed, with a minimum usage of 4 h/d over the period of four weeks (28 d). Circles represent individual participants (*N* = 16). One subject receiving a very low number of stimuli was subsequently excluded from analysis.

The wearable device had an inbuilt flash memory which logged the number of paired stimuli given every time it was switched on. Participants were asked to use the device for a minimum of 4 h/d, every day for four weeks (28 d). Based on the average stimulus frequency, this is equivalent to 268,800 paired stimuli. Figure 4 depicts each subject’s total number of stimuli received over the course of four weeks, with the dotted line indicating the target minimum stimulus number. Only three subjects exceeded the instructed minimum stimulus number; 12 subjects were below this level, but with a number judged acceptable. One subject had such a low compliance that they were subsequently excluded from group analysis.

**Figure 5.**
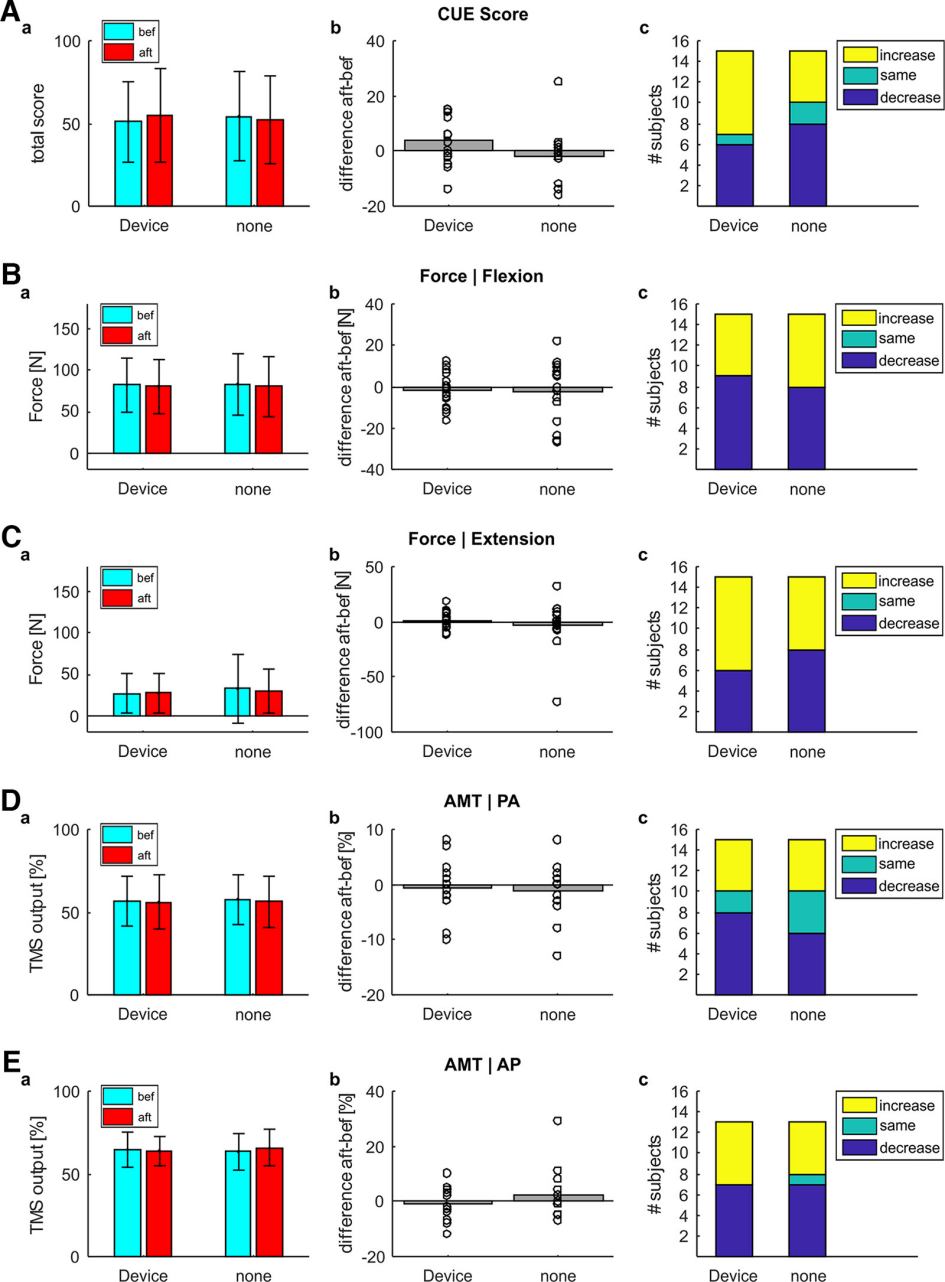
Group results for the SCI study. ***A***, Results for the CUE questionnaire (*N* = 15). The figure represents the total score achieved in the CUE questionnaire. ***a***, Total CUE score before (cyan) and after (red) four weeks of wearing the device (device) or four weeks of not using the device (none). Colored bars represent group means; error bars indicate SDs. ***b***, Difference in total CUE score between before and after using the device or not. A positive difference indicates an increase in total score after four weeks. Bars represent group means; circles show single subject values. ***c***, Number of subjects showing an increase (yellow) or decrease (blue) in total score after four weeks. ***B***, Results for the maximal force produced during elbow flexion (*N* = 15). The figure represents mean maximal force, measured with a force plate. Layout is the same as for ***A***. ***C***, Results for the maximal force produced during elbow extension (*N* = 15). The figure represents mean maximal force, measured with a force plate. Layout is the same as for ***A***. ***D***, Results for the active motor threshold of MEPs elicited with PA coil orientation (*N* = 15). The figure represents AMT. Layout is the same as for ***A***. ***E***, Results for the active motor threshold of MEPs elicited with AP coil orientation. For two subjects AMT was too high to detect AP MEPs (*N* = 13). The figure represents AMT. Layout is the same as for ***A***.

The total score achieved in the CUE questionnaire did not change between sessions (device *p* = 0.133, none *p* = 0.435; Fig. 5*Aa*) or between conditions (*p* = 0.177; Fig. 5*Ab*). Maximal force generated during elbow flexion (device *p* = 0.418, none *p* = 0.507; Fig. 5*B*) or extension (device *p* = 0.604, none *p* = 0.617; Fig. 5*C*) also remained unchanged and the differences did not vary across conditions (flexion *p* = 0.825, extension *p* = 0.471; Fig. 5*Bb*,*Cb*). Since participants had to come back on different days, we also compared AMT between sessions, but found no significant changes in stimulation threshold with either PA coil orientation (device *p* = 0.568, none *p* = 0.354; Fig. 5*D*) or AP coil orientation (device *p* = 0.630, none *p* = 0.419; Fig. 5*E*).

**Figure 6.**
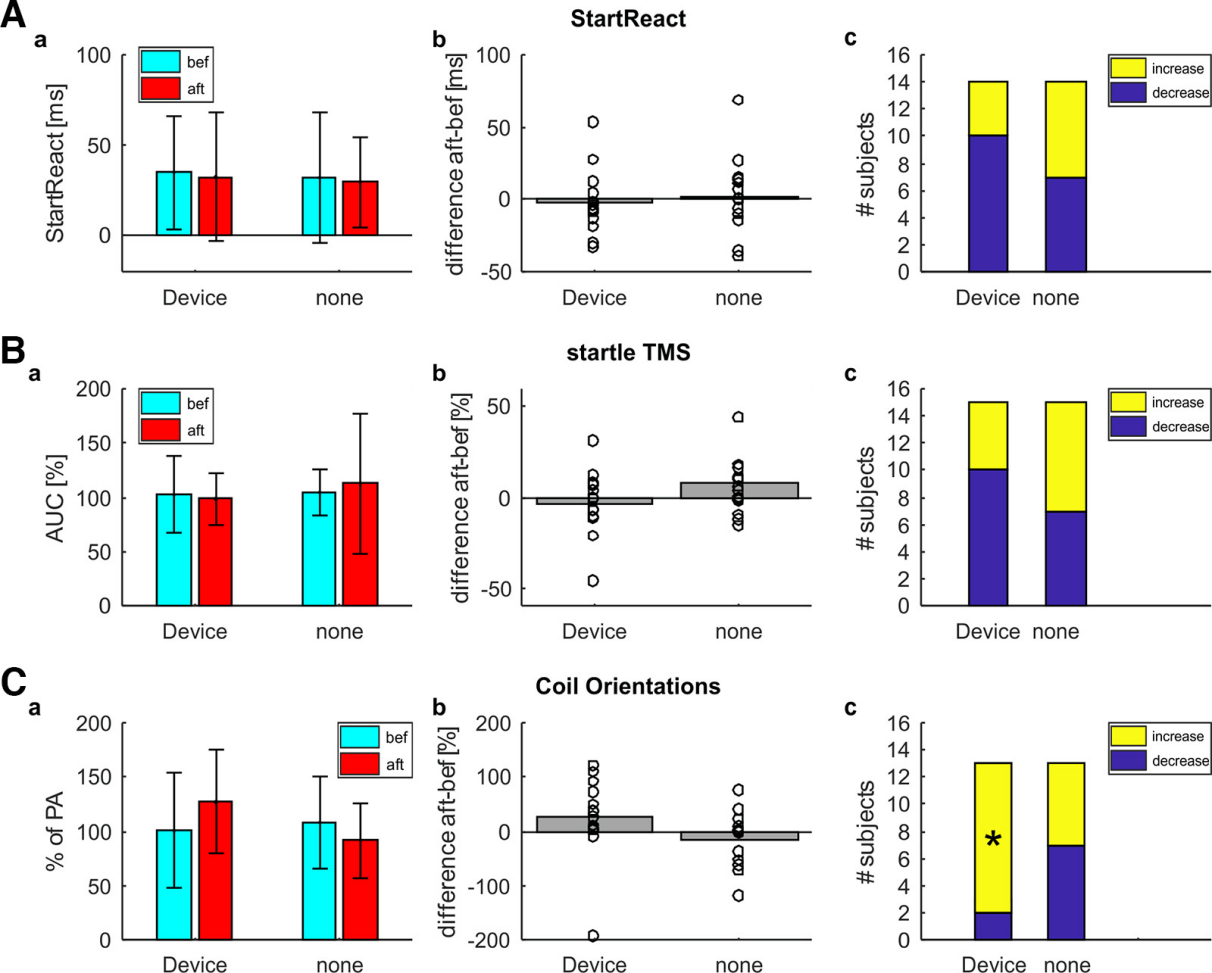
Group results for StartReact, startle TMS and MEPs with different coil orientations. ***A***, Results for the StartReact assessment. For one subject equipment failure prevented recordings, so subject had to be excluded for this assessment (*N* = 14). The figure represents StartReact effect (difference between VART and VSRT). ***a***, StartReact effect before (cyan) and after (red) wearing the device for four weeks (device) or four weeks of not using the device (none). Colored bars represent group means; error bars indicate SDs. ***b***, Difference in StartReact effect between before and after using the device or not. A positive difference indicates a more pronounced StartReact effect after four weeks. Bars represent group means; circles show single subject values. ***c***, Number of subjects showing an increase (yellow) or decrease (blue) in StartReact effect after four weeks. Asterisks indicate proportions significantly different from the 50% expected by chance. ***B***, Results for the conditioned MEPs with loud sounds (*N* = 15). The figure represents conditioned MEP amplitude normalized to unconditioned amplitude (as percentage of control). Layout is the same as for ***A***. ***C***, Results for the coil orientation assessment. For two subjects stimulation threshold was too high to detect AP MEPs, so subjects were excluded for the coil orientation assessment (*N* = 13). The figure represents MEP amplitudes elicited by AP orientation normalized to MEP amplitudes elicited by PA orientation. Layout is the same as for ***A***.

As with our healthy volunteers, there was no significant difference in StartReact effect after wearable device stimulation (device *p* = 0.461, none *p* = 0.476; Fig. 6*A*).

Figure 6*B* depicts group results for MEPs conditioned with loud sound. Notably, in these SCI subjects MEPs were not suppressed at baseline (device *p* = 0.796, none *p* = 0.4137; Fig. 6*Ba*), which is unexpected based on previous findings. MEP size remained unchanged across sessions (device *p* = 0.382, none *p* = 0.587) and did not differ between conditions (*p* = 0.471; Fig. 6*Bb*).

Figure 6*C* illustrates average size of MEPs elicited with AP coil orientation normalized to the size of MEPs elicited with PA coil orientation. There was no apparent difference in MEP size between sessions (device *p* = 0.246, none *p* = 0.281; Fig. 6*Ca*) or between conditions (*p* = 0.114; Fig. 6*Cb*). However, more subjects than expected by chance based on a binomial distribution showed an increase in MEP size elicited with AP compared with PA-induced currents after wearable device stimulation (device *p* = 0.022, none *p* = 0.500).

**Figure 7.**
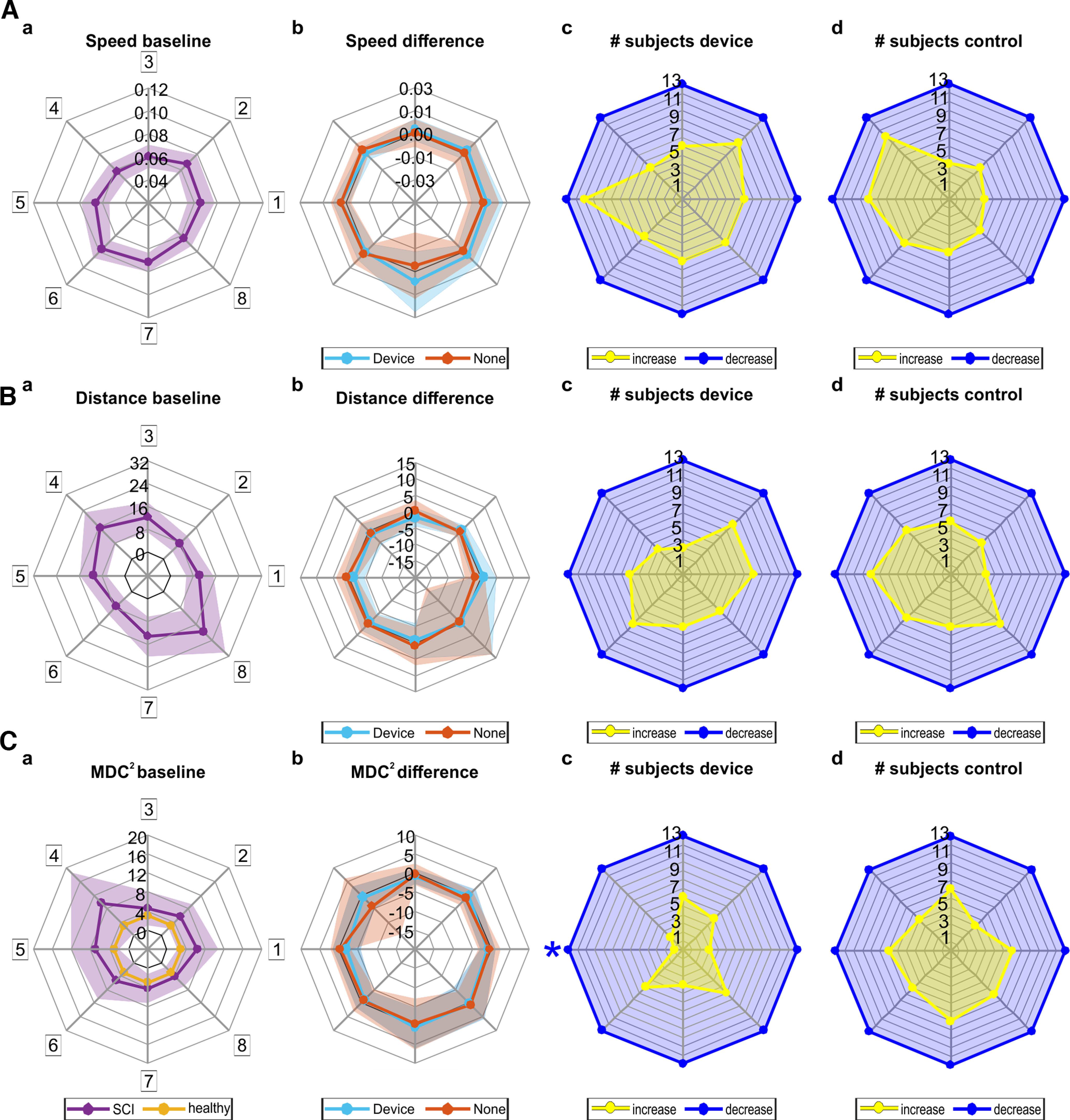
Group results for the planar reaching task. Two subjects were unable to perform the required reaching movements and group analysis is based on the remaining 13 participants. ***A***, Maximal speed of trajectories achieved while reaching for each target (Targets 1–8, boxed numbers). ***a***, Mean maximal speed (in m/s) of trajectories across SCI participants at baseline (*N* = 13). Purple line represents mean values, shaded area represents standard deviation. ***b***, Difference in maximal speed (in m/s) between before and after four weeks of using the device (light blue) or not (orange). ***c***, Number of subjects showing an increase (yellow) or decrease (blue) in maximal speed after four weeks of using the device. ***d***, Number of subjects showing an increase (yellow) or decrease (blue) in maximal speed after four weeks of not using the device. ***B***, Maximal distance from straight line (in mm) while reaching for each target (Targets 1–8). Layout is the same as for ***A***. ***C***, Mahalanobis distance squared (MDC^2^) of trajectories for each target (Targets 1–8). MDC^2^ was calculated by comparing trajectories from SCI participants (*N* = 13) with trajectories from healthy controls (*N* = 18) with functional PCA. ***a***, Mean MDC^2^ across SCI participants (purple) and healthy controls (amber). Layout is the same as for ***A***. Stars indicate proportions significantly different from the 50% expected by chance, for that target.

Group analysis of trajectories during the planar reaching task are shown in Figure 7. Targets are arranged radially according to where they were positioned during the task (for subjects using their right arm; targets were mirrored across the midline for subjects using their left arm), with target 7 being the closest to the subject’s body. Both maximal speed and distance from straight line were significantly modulated with target position (speed *F*_(7,84)_ = 8.648, *p* < 0.001, η^2^ = 0.419; distance *F*_(7,84)_ = 14.170, *p* < 0.001, η^2^ = 0.541; Fig. 7*Aa*,*Ba*). There was a significant effect of *target* for both measures after four weeks of using the device (speed *F*_(7,84)_ = 8.074, *p* < 0.001, η^2^ = 0.402; distance *F*_(7,84)_ = 16.092, *p* < 0.001, η^2^ = 0.573) and four weeks of not using the device (speed *F*_(7,84)_ = 8.205, *p* < 0.001, η^2^ = 0.406; distance *F*_(7,84)_ = 17.644, *p* < 0.001, η^2^ = 0.595), but no effect of *time* (speed “device” *F*_(1,12)_ = 3.759, *p* = 0.076, η^2^ = 0.239; speed “none” *F*_(1,12)_ = 0.101, *p* = 0.756, η^2^ = 0.008; distance “device” *F*_(1,12)_ = 0.439, *p* = 0.520, η^2^ = 0.035; distance “none” *F*_(7,84)_ = 17.644, *p* < 0.001, η^2^ = 0.595) or their interaction (speed “device” *F*_(7,84)_ = 0.627, *p* = 0.733, η^2^ = 0.050; speed “none” *F*_(7,84)_ = 0.808, *p* = 0.583, η^2^ = 0.063; distance “device” *F*_(7,84)_ = 0.395, *p* = 0.903, η^2^ = 0.032; distance “none” *F*_(7,84)_ = 0.333, *p* = 0.937, η^2^ = 0.027).

Comparing the differences between sessions (Fig. 7*Ab*,*Bb*) revealed no effect of condition (speed *F*_(1,12)_ = 0.743, *p* = 0.406, η^2^ = 0.058; distance *F*_(1,12)_ = 0.070, *p* = 0.797, η^2^ = 0.006), target (speed *F*_(7,84)_ = 0.547, *p* = 0.7961, η^2^ = 0.044; distance *F*_(7,84)_ = 0.462, *p* = 0.859, η^2^ = 0.037), or their interaction (speed *F*_(7,84)_ = 0.751, *p* = 0.629, η^2^ = 0.059; distance *F*_(7,84)_ = 0.340, *p* = 0.933, η^2^ = 0.028).

Trajectories were also evaluated using functional principal component analysis to compute Mahalanobis distance squared (MDC^2^; Fig. 7*C*) compared with trajectories from healthy volunteers (*N* = 18). A mixed ANOVA with repeated measure target (1–8) and between-subject factor group (SCI, healthy) was used to analyze trajectories at baseline (Fig. 7*Ca*). Mauchly’s Test of Sphericity indicated that the assumption of sphericity had been violated (χ^2^(27) = 239.232, *p* < 0.001) and therefore a Greenhouse–Geisser correction was used. Overall quality of trajectories from SCI participants differed significantly from healthy trajectories at baseline, with a main effect of group (*F*_(1,29)_ = 15.686, *p* < 0.001, η^2^ = 0.351), target (*F*_(7,203)_ = 3.518, *p* = 0.043, η^2^ = 0.108) and their interaction (*F*_(7,203)_ = 3.518, *p* = 0.0431, η^2^ = 0.108).

However, there was no effect of time (device *F*_(1,12)_ = 3.329, *p* = 0.093, η^2^ = 0.217; none *F*_(1,12)_ = 2.005, *p* = 0.182, η^2^ = 0.143), target (device *F*_(7,84)_ = 1.779, *p* = 0.102, η^2^ = 0.129; none *F*_(7,84)_ = 2.138, *p* = 0.048, η^2^ = 0.151), or their interaction (device *F*_(7,84)_ = 0.651, *p* = 0.713, η^2^ = 0.051; none *F*_(7,84)_ = 1.009, *p* = 0.431, η^2^ = 0.078) on MDC^2^ across sessions. Similar to maximal speed and distance to straight line, there was no effect of condition (*F*_(1,12)_ = 0.091, *p* = 0.768, η^2^ = 0.008), target (*F*_(7,84)_ = 1.803, *p* = 0.097, η^2^ = 0.131), or their interaction (*F*_(7,84)_ = 0.521, *p* = 0.817, η^2^ = 0.042) on MDC^2^ when looking at the differences between sessions (Fig. 7*Cb*).

Significantly more subjects than expected by chance showed a decrease in MDC^2^ after wearable device stimulation for target 5 (device *p* = 0.004, none *p* = 0.710) and target 4 (device *p* = 0.022, none *p* = 0.290), although target four was not significant after correction for multiple comparisons.

## Discussion

Unlike our previous results with the device targeting the biceps muscle (Germann and Baker, 2021), pairing electrical stimulation to the triceps muscle with auditory clicks for 6 h did not lead to changes in motor output in healthy volunteers.

A study by Foysal and Baker (2019) demonstrated that intrinsic hand and flexor muscle have a higher potential to show plasticity than extensors, which could explain the differences in our results. Although extensors might remain unaffected by our 6-h protocol, it remained possible that prolonged stimulation over several weeks might be enough to induce plasticity. However, there also were no significant electrophysiological or functional changes in triceps after four weeks of paired stimulation in chronic cervical SCI.

### Contrast with previous work: triceps does not show plasticity

Increased amplitude in MEPs elicited by AP currents, increased StartReact effect and reduction in MEP suppression following loud auditory stimuli were previously all observed in the biceps muscle after wearable device stimulation for 6 h (Germann and Baker, 2021); all of those changes point toward subcortical effects, such as enhanced RST output to the biceps motor nucleus.

Because the overt startle reflex is known to involve the reticulospinal tract (Davis et al., 1982), several studies suggest that the size of the StartReact effect is a measure of RST outflow (Valls-Solé et al., 1999; Carlsen et al., 2004; Rothwell, 2006; Baker and Perez, 2017; Smith et al., 2019); this has been recently confirmed by studies in monkeys from this laboratory (Tapia et al., 2022).

The cortical suppression of MEPs following LAS most likely results from stimulation of the reticular formation by the loud sound (Hammond, 1973; Leitner et al., 1980; Davis et al., 1982; Fisher et al., 2012; Tapia et al., 2022), and activation of cortical interneurons via reticulo-thalamic projections (Pare et al., 1988; Steriade et al., 1988).

TMS can preferentially recruit different descending waves in humans by changing the direction of the current induced in the brain (Sakai et al., 1997; Ziemann and Rothwell, 2000). PA-directed current predominantly recruits early, whereas AP current predominantly recruits late I-waves (Day et al., 1989; Sakai et al., 1997; Di Lazzaro et al., 2001; Ni et al., 2011), which may be more heavily modulated by subcortical sources (Tokimura et al., 1996; Ziemann et al., 1998).

None of these measures showed any significant changes for the triceps muscle after paired stimulation in healthy volunteers or after prolonged wearable device usage in chronic cervical SCI. In addition to our electrophysiological measurements, we also had a range of functional measurements to assess motor function before and after wearable device stimulation in participants with chronic cervical SCI. For one, we assessed maximal force produced before and after wearing the device (Fig. 5*B*,*C*), although this too remained unchanged in both biceps and triceps muscle.

In addition to strength, an important aspect of motor control is skilled muscle use; this is harder to assess as most movements have significant antigravity strength requirements. In order to separate motor control of the upper limb from strength, we used a planar reaching task similar to previous studies in stroke (Goldsmith and Kitago, 2016; Cortes et al., 2017). This task allowed us to compare range, speed, and accuracy of upper limb movements across sessions, which overall did not change significantly.

In our previous study, auditory clicks were always delivered contralaterally to the target muscle. However, it has been shown that galvanic-evoked vestibulocollic reflexes project contralaterally to flexors and ipsilaterally to extensors (Uchino et al., 1997; Watson and Colebatch, 1998a). Auditory clicks of 100 dB are able to evoke vestibulospinal reflex responses with a similar latency as galvanic stimulation (Watson and Colebatch, 1998b). Therefore, when targeting the elbow extensor triceps brachii, auditory clicks might need to be delivered ipsilaterally. For comparison, half of our healthy participants wore the earpiece in the contralateral ear and half in the ipsilateral ear. As there were no significant differences between these groups and because of the low number of participants we also combined the results (Fig. 2).

Although reticulospinal neurons may respond better to plasticity protocols than corticospinal neurons (Vavrek et al., 2007; Zörner et al., 2014), there is a hierarchy in the ability to induce plastic changes across muscle groups (Foysal and Baker, 2019), with extensor muscles showing a much lower potential for plasticity. In fact, the study by Foysal and Baker (2019) showed that even when extensor muscles were stimulated, outputs were enhanced to flexors but not extensors. However, this difference was only seen in protocols that require integration of sensory input, while the same level of plasticity was observed when using a rTMS protocol. There is a similar difference in the ability to induce plasticity between muscles in the lower limb. The plantar flexor muscle soleus does not show plastic changes in the corticospinal tract after neuromuscular electrical stimulation (Lagerquist et al., 2012), whereas the dorsiflexor tibealis anterior does (Khaslavskaia and Sinkjaer, 2005).

This might be because flexor and extensor muscles receive differential control from corticospinal and reticulospinal pathways. Through corticospinal connections, flexors might receive larger monosynaptic facilitation and extensors stronger disynaptic inhibition in both monkeys (Phillips and Porter, 1964) and humans (Palmer and Ashby, 1992), although other work has suggested no flexor-extensor difference (Park et al., 2004). Stimulation of the reticular formation in monkeys typically evokes bilateral responses, but the prevalent pattern is ipsilateral extensor suppression and flexor facilitation with the opposite pattern occurring contralaterally (Davidson and Buford, 2006; Herbert et al., 2010).

After contralateral CST lesion in monkey, there was no evidence of novel connections from ipsilateral corticospinal fibers that would make any significant contribution to recovery (Zaaimi et al., 2012). In contrast, there was a nonuniform strengthening of brainstem connections, with inputs to forearm flexor and intrinsic hand motorneurons enhanced, whereas connections to extensors remained unchanged (Zaaimi et al., 2012). Limited CST connections and enhanced RST output has been associated with spasticity in SCI survivors (Sangari and Perez, 2019). Whether reticulospinal reorganization after injury is helpful or detrimental remains a topic of discussion (Karbasforoushan et al., 2019), but many animal models of SCI have provided evidence that it can contribute to functional tasks (Wakabayashi et al., 2001; Ballermann and Fouad, 2006; Filli et al., 2014; May et al., 2017).

Importantly, there is no evidence of increased spasticity in stroke survivors using the wearable device (Choudhury et al., 2020). There were likewise no adverse events of any type reported during our study with SCI participants.

All of the above evidence suggests that reticulospinal inputs compensate for the loss of corticospinal axons, but may do so preferentially in flexors, which could explain the asymmetrical recovery of biceps and triceps muscles observed in people after SCI (Sangari and Perez, 2020). The failure to enhance reticulospinal inputs to triceps could also explain why our plasticity protocol leads to significantly enhanced motor output in biceps but not triceps. However, it should be noted that given our small number of participants in both studies, we cannot rule out a possible small effect. Choudhury et al. (2020) showed a significant improvement in hand function in stroke patients after four weeks of wearable device usage with a protocol that targeted forearm extensors; however, the range of motion and maximum contraction torque about the wrist did not change. It is possible therefore that the benefits in that study were more nonspecific, rather than resulting from a direct effect in the extensor muscles. There is also the possibility that there is an inherent difference between distal forearm extensors and the more proximal elbow extensor, something that would need to be investigated in future studies.

### Study limitations

Compliance was variable between SCI participants (Fig. 4), with only three participants receiving more than the instructed minimum number of stimuli and 13 receiving fewer. Choudhury et al. (2020) demonstrated that the extent of functional gain in stroke patients was correlated with stimulus number. The smaller number of participants in our study, and even fewer participants reaching minimum compliance, could have affected our results.

The CUE questionnaire is designed to measure upper extremity functional limitations in individuals with tetraplegia. However, 12 out of 32 questions focus on hand and finger function, hardly something expected to change after our plasticity protocol targeting the triceps.

Furthermore, we recruited participants with both complete and incomplete cervical SCI. Even when voluntary movement is completely absent on clinical evaluation, neurophysiological methods using surface electromyography are sometimes able to identify and quantify evidence of preserved translesional conduction in chronic clinically complete SCI subjects (McKay et al., 2004, 2011). However, each SCI lesion is highly individualized in regards to severity of impairment and which particular descending tracts are spared (Kakulas, 2004) and including a wider range of participants must invariably introduce more variability in the data. This considerable diversity in pathway interruption would also affect the amount of RST damage the study participants might have sustained.

The heterogeneity of the participants was mostly because of the lack of injury-specific inclusion criteria (see Methods), as anyone with a cervical injury was allowed to participate. There was also very little clinical baseline data available, further contributing to the diverse nature of the participants. Future more extensive studies could use, for example, the ASIA scale to stratify participants and possibly improve the chance to detect an effect.

## Synthesis

Reviewing Editor: David Schoppik, New York University Decisions are customarily a result of the Reviewing Editor and the peer reviewers coming together and discussing their recommendations until a consensus is reached. When revisions are invited, a fact-based synthesis statement explaining their decision and outlining what is needed to prepare a revision will be listed below. The following reviewer(s) agreed to reveal their identity: Nick Donaldson.

Commentary

We agreed that this was an interesting paper with negative results: the device does not improve control over the elbow extensors in the same way as previously shown for the elbow flexors. The authors put it like this at the end of the Introduction: “triceps may be more resistant to stimulation protocols which induce plasticity in sub-cortical pathways”. We considered what this might actually mean:

We interpreted the method as paired associative stimulation where stimulation at two sites, such as TMS to the motor cortex and electrical stimulation to a muscle, cause volleys of action potentials to arrive almost simultaneously, for example at the anterior horn cells, strengthening the synapses between the upper and lower motor neurons. In this work, the idea is to utilise the reticulospinal tract neurons because those in the corticospinal tract may be damaged by SCI. The aim is to strengthen synapses in the brainstem (line 316). Electrical stimulation was applied to the triceps surae 10ms before an auditory click to the contralateral ear, so that the afferent volley from the muscle and the auditory volley to the reticulospinal tract neurons are coincident. The reticulospinal tract provides the motor reflex responses to sounds and therefore generally connects to the lower motor neurons and the muscles. In the previous work, the authors showed that such reinforcement improves voluntary control (i.e. from the motor cortex) to the elbow flexors but here they found that it does not improve voluntary control of the elbow extensors. What is the difference? Are not there two possibilities: either the synaptic weights are not changing or because there is not a pathway from the cortex to the RST for the extensors, though there is for the flexors? Our understanding of the Discussion on page 26 is that the authors infer that the latter is the reason (“lack of extra inputs to triceps”). Perhaps it could be made clearer that these “extra inputs” are triceps inputs to the RST in the brainstem?

We found it interesting to compare this paper with Lagerquist et al (Exp Brain Research, 2012, 222:41-53) who measured cortico-spinal excitability in able bodied people using TMS and evoked potentials, following voluntary contractions or muscle stimulation or both together. For dorsiflexors of the ankle, all three increase excitability whereas for the soleus (plantarflexor), none did so. These subjects had intact cortical-spinal tracts but still the extensors did not show neuroplastic changes. There is interesting discussion in this paper about the reasons for the differences.

Possible Improvements

The Methods section is long because of the two experiments, the AB and the SCI studies. It would be helpful to add a table near the beginning of the section which shows which methods were used in each study. Having seen the table at the start, the reader will find it easier to understand the relevance of all the various methods.

TMS Section. Line 168: is the 45{degree sign} lateral or medial?

Line 170, definition of hotspot: “where the largest MEP in the triceps muscle could be evoked with the minimum intensity (Rothwell, 1999)”. This seems a poor definition; is it the position of the largest MEP or the minimum intensity? Can it be explained better by saying how it is found?

Line 185. Would “reaction time to EMG” by better?

Line 241. Please add “the” after “contralateral hemisphere in”.

Line 258. Two sentences are almost repetition.

Line 324. Please describe the averaging filter.

Figure 1. We did not understand this set-up from the diagram. If the target LEDs are in layer f (pointing upwards) while LED c, on the sled, shines a spot of light up at layer f. But what is the subject looking at? If she were looking at a sloping mirror, it would make sense but e appears to be horizontal?

Lines 530-531 were there differences?

Line 576 please drop “very”

## Author Response

Manuscript Instructions

- One page 5 (around line 99), please remove “Faculty of Medical Sciences at Newcastle University.” To facilitate the double-blind review process, no information may be present that will reveal the identity of any author.

The information has been removed.

---------------------------------------------

Synthesis of Reviews:

Computational Neuroscience Model Code Accessibility Comments for Author (Required):

N/A

Synthesis Statement for Author (Required):

Commentary

We agreed that this was an interesting paper with negative results: the device does not improve control over the elbow extensors in the same way as previously shown for the elbow flexors. The authors put it like this at the end of the Introduction: “triceps may be more resistant to stimulation protocols which induce plasticity in sub-cortical pathways”. We considered what this might actually mean:

We interpreted the method as paired associative stimulation where stimulation at two sites, such as TMS to the motor cortex and electrical stimulation to a muscle, cause volleys of action potentials to arrive almost simultaneously, for example at the anterior horn cells, strengthening the synapses between the upper and lower motor neurons. In this work, the idea is to utilise the reticulospinal tract neurons because those in the corticospinal tract may be damaged by SCI. The aim is to strengthen synapses in the brainstem (line 316). Electrical stimulation was applied to the triceps surae 10ms before an auditory click to the contralateral ear, so that the afferent volley from the muscle and the auditory volley to the reticulospinal tract neurons are coincident. The reticulospinal tract provides the motor reflex responses to sounds and therefore generally connects to the lower motor neurons and the muscles. In the previous work, the authors showed that such reinforcement improves voluntary control (i.e. from the motor cortex) to the elbow flexors but here they found that it does not improve voluntary control of the elbow extensors. What is the difference? Are not there two possibilities: either the synaptic weights are not changing or because there is not a pathway from the cortex to the RST for the extensors, though there is for the flexors? Our understanding of the Discussion on page 26 is that the authors infer that the latter is the reason (“lack of extra inputs to triceps”). Perhaps it could be made clearer that these “extra inputs” are triceps inputs to the RST in the brainstem?

We have now reworded this sentence to improve clarity.

We found it interesting to compare this paper with Lagerquist et al (Exp Brain Research, 2012, 222:41-53) who measured cortico-spinal excitability in able bodied people using TMS and evoked potentials, following voluntary contractions or muscle stimulation or both together. For dorsiflexors of the ankle, all three increase excitability whereas for the soleus (plantarflexor), none did so. These subjects had intact cortical-spinal tracts but still the extensors did not show neuroplastic changes. There is interesting discussion in this paper about the reasons for the differences.

Thank you for alerting us to this highly relevant paper, which we have now cited.

Possible Improvements

The Methods section is long because of the two experiments, the AB and the SCI studies. It would be helpful to add a table near the beginning of the section which shows which methods were used in each study. Having seen the table at the start, the reader will find it easier to understand the relevance of all the various methods.

A table summarizing assessments used in each of the studies has been added.

TMS Section. Line 168: is the 45deg lateral or medial?

Lateral. This has now been included in the original sentence.

Line 170, definition of hotspot: “where the largest MEP in the triceps muscle could be evoked with the minimum intensity (Rothwell, 1999)”. This seems a poor definition; is it the position of the largest MEP or the minimum intensity? Can it be explained better by saying how it is found?

The hotspot is conventionally defined as the location over which TMS evokes the biggest MEPs in the target muscle. The definition in the methods has been adjusted to avoid confusion.

Line 185. Would “reaction time to EMG” by better?

We are measuring the reaction time from the recorded EMG, meaning EMG onset is taken as the reaction time. It’s the reaction time to the light, or the light with the sound, as measured by EMG onset. The sentence has been reworded to make this intended meaning clearer.

Line 241. Please add “the” after “contralateral hemisphere in”.

This has been added.

Line 258. Two sentences are almost repetition.

The information has been collated into one sentence.

Line 324. Please describe the averaging filter.

The EMG traces were rectified and then averaged; this has been stated. No further filtering (in the sense of limiting frequencies present in the signals) was used.

Figure 1. We did not understand this set-up from the diagram. If the target LEDs are in layer f (pointing upwards) while LED c, on the sled, shines a spot of light up at layer f. But what is the subject looking at? If she were looking at a sloping mirror, it would make sense but e appears to be horizontal?

Layer f is a two-way mirror, as indicated in the figure legend. This means that the LEDs from layer e are mirrored down onto layer f so the subject can see them. But the subject can also see the sled and LED c being projected up as it is a two-way mirror and therefore see-through.

Lines 530-531 were there differences?

There were no significant differences between the contralateral or ipsilateral ear. This has now been stated clearly in the results.

Line 576 please drop “very”

This has been dropped as requested.

## References

[B1] Angeli CA, Edgerton VR, Gerasimenko YP, Harkema SJ (2014) Altering spinal cord excitability enables voluntary movements after chronic complete paralysis in humans. Brain 137:1394–1409. 10.1093/brain/awu038 24713270 PMC3999714

[B2] Arai N, Okabe S, Furubayashi T, Terao Y, Yuasa K, Ugawa Y (2005) Comparison between short train, monophasic and biphasic repetitive transcranial magnetic stimulation (rTMS) of the human motor cortex. Clin Neurophysiol 116:605–613. 10.1016/j.clinph.2004.09.020 15721074

[B3] Baker SN, Perez MA (2017) Reticulospinal contributions to gross hand function after human spinal cord injury. J Neurosci 37:9778–9784. 10.1523/JNEUROSCI.3368-16.2017 28871033 PMC5628413

[B4] Ballermann M, Fouad K (2006) Spontaneous locomotor recovery in spinal cord injured rats is accompanied by anatomical plasticity of reticulospinal fibers. Eur J Neurosci 23:1988–1996. 10.1111/j.1460-9568.2006.04726.x 16630047

[B5] Barthelemy D, Willerslev-Olsen M, Lundell H, Biering-Sorensen F, Nielsen JB (2015) Assessment of transmission in specific descending pathways in relation to gait and balance following spinal cord injury. Prog Brain Res 218:79–101. 25890133 10.1016/bs.pbr.2014.12.012

[B6] Belhaj-Saïf A, Cheney PD (2000) Plasticity in the distribution of the red nucleus output to forearm muscles after unilateral lesions of the pyramidal tract. J Neurophysiol 83:3147–3153. 10.1152/jn.2000.83.5.3147 10805709

[B7] Benavides FD, Jo HJ, Lundell H, Edgerton VR, Gerasimenko Y, Perez MA (2020) Cortical and subcortical effects of transcutaneous spinal cord stimulation in humans with tetraplegia. J Neurosci 40:2633–2643. 10.1523/JNEUROSCI.2374-19.2020 31996455 PMC7096150

[B8] Benjamini Y, Hochberg Y (1995) Controlling the false discovery rate - a practical and powerful approach to multiple testing. J R Stat Soc Series B Stat Methodol 57:289–300. 10.1111/j.2517-6161.1995.tb02031.x

[B9] Calancie B, Molano MR, Broton JG (2004) EMG for assessing the recovery of voluntary movement after acute spinal cord injury in man. Clin Neurophysiol 115:1748–1759. 10.1016/j.clinph.2004.03.002 15261853

[B10] Carlsen AN, Chua R, Inglis JT, Sanderson DJ, Franks IM (2004) Can prepared responses be stored subcortically? Exp Brain Res 159:301–309. 10.1007/s00221-004-1924-z 15480608

[B11] Cauraugh J, Light K, Kim S, Thigpen M, Behrman A (2000) Chronic motor dysfunction after stroke: recovering wrist and finger extension by electromyography-triggered neuromuscular stimulation. Stroke 31:1360–1364. 10.1161/01.str.31.6.1360 10835457

[B12] Choudhury S, Shobhana A, Singh R, Sen D, Anand SS, Shubham S, Baker MR, Kumar H, Baker SN (2019) The relationship between enhanced reticulospinal outflow and upper limb function in chronic stroke patients. Neurorehabil Neural Repair 33:375–383. 10.1177/1545968319836233 30913964

[B13] Choudhury S, Singh R, Shobhana A, Sen D, Anand SS, Shubham S, Gangopadhyay S, Baker MR, Kumar H, Baker SN (2020) A novel wearable device for motor recovery of hand function in chronic stroke survivors. Neurorehabil Neural Repair 34:600–608. 10.1177/1545968320926162 32452275 PMC8207486

[B14] Cortes JC, Goldsmith J, Harran MD, Xu J, Kim N, Schambra HM, Luft AR, Celnik P, Krakauer JW, Kitago T (2017) A short and distinct time window for recovery of arm motor control early after stroke revealed with a global measure of trajectory kinematics. Neurorehabil Neural Repair 31:552–560. 10.1177/1545968317697034 28506149 PMC5434710

[B15] Davidson AG, Buford JA (2006) Bilateral actions of the reticulospinal tract on arm and shoulder muscles in the monkey: stimulus triggered averaging. Exp Brain Res 173:25–39. 10.1007/s00221-006-0374-1 16506008

[B16] Davis M, Gendelman DS, Tischler MD, Gendelman PM (1982) A primary acoustic startle circuit: lesion and stimulation studies. J Neurosci 2:791–805. 10.1523/JNEUROSCI.02-06-00791.1982 7086484 PMC6564345

[B17] Day BL, Dressler D, Maertens de Noordhout A, Marsden CD, Nakashima K, Rothwell JC, Thompson PD (1989) Electric and magnetic stimulation of human motor cortex: surface EMG and single motor unit responses. J Physiol 412:449–473. 10.1113/jphysiol.1989.sp017626 2489409 PMC1190586

[B18] Dean LR, Baker SN (2017) Fractionation of muscle activity in rapid responses to startling cues. J Neurophysiol 117:1713–1719. 10.1152/jn.01009.2015 28003416 PMC5384977

[B19] Di Lazzaro V, Oliviero A, Saturno E, Pilato F, Insola A, Mazzone P, Profice P, Tonali P, Rothwell JC (2001) The effect on corticospinal volleys of reversing the direction of current induced in the motor cortex by transcranial magnetic stimulation. Exp Brain Res 138:268–273. 10.1007/s002210100722 11417469

[B20] Ditunno JF Jr, Stover SL, Freed MM, Ahn JH (1992) Motor recovery of the upper extremities in traumatic quadriplegia: a multicenter study. Arch Phys Med Rehabil 73:431–436. 1580769

[B21] Ditunno JF Jr, Cohen ME, Hauck WW, Jackson AB, Sipski ML (2000) Recovery of upper-extremity strength in complete and incomplete tetraplegia: a multicenter study. Arch Phys Med Rehabil 81:389–393. 10.1053/mr.2000.3779 10768525

[B22] Filli L, Engmann AK, Zörner B, Weinmann O, Moraitis T, Gullo M, Kasper H, Schneider R, Schwab ME (2014) Bridging the gap: a reticulo-propriospinal detour bypassing an incomplete spinal cord injury. J Neurosci 34:13399–13410. 10.1523/JNEUROSCI.0701-14.2014 25274818 PMC6608315

[B23] Fisher KM, Zaaimi B, Baker SN (2012) Reticular formation responses to magnetic brain stimulation of primary motor cortex. J Physiol 590:4045–4060. 10.1113/jphysiol.2011.226209 22674723 PMC3464356

[B24] Foysal KMR, Baker SN (2019) A hierarchy of corticospinal plasticity in human hand and forearm muscles. J Physiol 597:2729–2739. 10.1113/JP277462 30839110 PMC6567854

[B25] Foysal KM, de Carvalho F, Baker SN (2016) Spike timing-dependent plasticity in the long-latency stretch reflex following paired stimulation from a wearable electronic device. J Neurosci 36:10823–10830. 10.1523/JNEUROSCI.1414-16.2016 27798137 PMC5083010

[B26] Furubayashi T, Ugawa Y, Terao Y, Hanajima R, Sakai K, Machii K, Mochizuki H, Shiio Y, Uesugi H, Enomoto H, Kanazawa I (2000) The human hand motor area is transiently suppressed by an unexpected auditory stimulus. Clin Neurophysiol 111:178–183. 10.1016/s1388-2457(99)00200-x 10656526

[B27] Germann M, Baker SN (2021) Evidence for subcortical plasticity after paired stimulation from a wearable device. J Neurosci 41:1418–1428. 10.1523/JNEUROSCI.1554-20.2020 33441436 PMC7896019

[B28] Goldsmith J, Kitago T (2016) Assessing systematic effects of stroke on motorcontrol by using hierarchical function-on-scalar regression. J R Stat Soc Ser C Appl Stat 65:215–236. 10.1111/rssc.12115 27546913 PMC4988692

[B29] Hammond GR (1973) Lesions of pontine and medullary reticular formation and prestimulus inhibition of the acoustic startle reaction in rats. Physiol Behav 10:239–243. 10.1016/0031-9384(73)90304-1 4575307

[B30] Herbert WJ, Davidson AG, Buford JA (2010) Measuring the motor output of the pontomedullary reticular formation in the monkey: do stimulus-triggered averaging and stimulus trains produce comparable results in the upper limbs? Exp Brain Res 203:271–283. 10.1007/s00221-010-2231-5 20379705 PMC2923487

[B31] Honeycutt CF, Kharouta M, Perreault EJ (2013) Evidence for reticulospinal contributions to coordinated finger movements in humans. J Neurophysiol 110:1476–1483. 10.1152/jn.00866.2012 23825395 PMC4042417

[B32] Irvine DR, Jackson GD (1983) Auditory input to neurons in mesencephalic and rostral pontine reticular formation: an electrophysiological and horseradish peroxidase study in the cat. J Neurophysiol 49:1319–1333. 10.1152/jn.1983.49.6.1319 6875625

[B33] Kakulas BA (1999) A review of the neuropathology of human spinal cord injury with emphasis on special features. J Spinal Cord Med 22:119–124. 10.1080/10790268.1999.11719557 10826269

[B34] Kakulas BA (2004) Neuropathology: the foundation for new treatments in spinal cord injury. Spinal Cord 42:549–563. 10.1038/sj.sc.3101670 15346131

[B35] Kamper DG, Harvey RL, Suresh S, Rymer WZ (2003) Relative contributions of neural mechanisms versus muscle mechanics in promoting finger extension deficits following stroke. Muscle Nerve 28:309–318. 10.1002/mus.10443 12929190

[B36] Karbasforoushan H, Cohen-Adad J, Dewald JPA (2019) Brainstem and spinal cord MRI identifies altered sensorimotor pathways post-stroke. Nat Commun 10:3524. 10.1038/s41467-019-11244-3 31388003 PMC6684621

[B37] Khaslavskaia S, Sinkjaer T (2005) Motor cortex excitability following repetitive electrical stimulation of the common peroneal nerve depends on the voluntary drive. Exp Brain Res 162:497–502. 10.1007/s00221-004-2153-1 15702321

[B38] Kitago T, Goldsmith J, Harran M, Kane L, Berard J, Huang S, Ryan SL, Mazzoni P, Krakauer JW, Huang VS (2015) Robotic therapy for chronic stroke: general recovery of impairment or improved task-specific skill? J Neurophysiol 114:1885–1894. 10.1152/jn.00336.2015 26180120 PMC4575974

[B39] Lagerquist O, Mang CS, Collins DF (2012) Changes in spinal but not cortical excitability following combined electrical stimulation of the tibial nerve and voluntary plantar-flexion. Exp Brain Res 222:41–53. 10.1007/s00221-012-3194-5 22899312

[B40] Lawrence DG, Kuypers HG (1968a) The functional organization of the motor system in the monkey. I. The effects of bilateral pyramidal lesions. Brain 91:1–14. 10.1093/brain/91.1.1 4966862

[B41] Lawrence DG, Kuypers HG (1968b) The functional organization of the motor system in the monkey. II. The effects of lesions of the descending brain-stem pathways. Brain 91:15–36. 10.1093/brain/91.1.15 4966860

[B42] Leiras R, Velo P, Martín-Cora F, Canedo A (2010) Processing afferent proprioceptive information at the main cuneate nucleus of anesthetized cats. J Neurosci 30:15383–15399. 10.1523/JNEUROSCI.2193-10.2010 21084595 PMC6633671

[B43] Leitner DS, Powers AS, Hoffman HS (1980) The neural substrate of the startle response. Physiol Behav 25:291–297. 10.1016/0031-9384(80)90219-x 7413836

[B44] Marino RJ, Shea JA, Stineman MG (1998) The capabilities of upper extremity instrument: reliability and validity of a measure of functional limitation in tetraplegia. Arch Phys Med Rehabil 79:1512–1521. 10.1016/s0003-9993(98)90412-9 9862292

[B45] May Z, Fenrich KK, Dahlby J, Batty NJ, Torres-Espín A, Fouad K (2017) Following spinal cord injury transected reticulospinal tract axons develop new collateral inputs to spinal interneurons in parallel with locomotor recovery. Neural Plast 2017:1932875. 10.1155/2017/1932875 29138697 PMC5613456

[B46] McKay WB, Lim HK, Priebe MM, Stokic DS, Sherwood AM (2004) Clinical neurophysiological assessment of residual motor control in post-spinal cord injury paralysis. Neurorehabil Neural Repair 18:144–153. 10.1177/0888439004267674 15375274

[B47] McKay WB, Ovechkin AV, Vitaz TW, Terson de Paleville DG, Harkema SJ (2011) Neurophysiological characterization of motor recovery in acute spinal cord injury. Spinal Cord 49:421–429. 10.1038/sc.2010.145 21079622 PMC3444805

[B48] Nathan PW, Smith MC (1955) Long descending tracts in man. I. Review of present knowledge. Brain 78:248–303. 10.1093/brain/78.2.248 13239911

[B49] Ni Z, Charab S, Gunraj C, Nelson AJ, Udupa K, Yeh IJ, Chen R (2011) Transcranial magnetic stimulation in different current directions activates separate cortical circuits. J Neurophysiol 105:749–756. 10.1152/jn.00640.2010 21148098

[B50] Onodera S, Hicks TP (2010) Carbocyanine dye usage in demarcating boundaries of the aged human red nucleus. PLoS One 5:e14430. 10.1371/journal.pone.0014430 21203458 PMC3009723

[B51] Palmer E, Ashby P (1992) Corticospinal projections to upper limb motoneurones in humans. J Physiol 448:397–412. 10.1113/jphysiol.1992.sp019048 1593472 PMC1176206

[B52] Pare D, Smith Y, Parent A, Steriade M (1988) Projections of brainstem core cholinergic and non-cholinergic neurons of cat to intralaminar and reticular thalamic nuclei. Neuroscience 25:69–86. 10.1016/0306-4522(88)90007-3 3393287

[B53] Park MC, Belhaj-Saïf A, Cheney PD (2004) Properties of primary motor cortex output to forelimb muscles in rhesus macaques. J Neurophysiol 92:2968–2984. 10.1152/jn.00649.2003 15163675

[B54] Petersen JA, Wilm BJ, von Meyenburg J, Schubert M, Seifert B, Najafi Y, Dietz V, Kollias S (2012) Chronic cervical spinal cord injury: DTI correlates with clinical and electrophysiological measures. J Neurotrauma 29:1556–1566. 10.1089/neu.2011.2027 22150011

[B55] Phillips CG, Porter R (1964) The pyramidal projection to motoneurones of some muscle groups of the baboon’s forelimb. Prog Brain Res 12:222–245. 14202441 10.1016/s0079-6123(08)60625-1

[B56] Rosengren SM, Welgampola MS, Colebatch JG (2010) Vestibular evoked myogenic potentials: past, present and future. Clin Neurophysiol 121:636–651. 10.1016/j.clinph.2009.10.016 20080441

[B57] Rosenzweig ES, Courtine G, Jindrich DL, Brock JH, Ferguson AR, Strand SC, Nout YS, Roy RR, Miller DM, Beattie MS, Havton LA, Bresnahan JC, Edgerton VR, Tuszynski MH (2010) Extensive spontaneous plasticity of corticospinal projections after primate spinal cord injury. Nat Neurosci 13:1505–1510. 10.1038/nn.2691 21076427 PMC3144760

[B58] Rothwell JC (2006) The startle reflex, voluntary movement, and the reticulospinal tract. Suppl Clin Neurophysiol 58:223–231. 16623334 10.1016/s1567-424x(09)70071-6

[B82] Rothwell JC, Hallett M, Berardelli A, Eisen A, Rossini P, Paulus W (1999) Magnetic stimulation: motor evoked potentials. Electroencephalogr Clin Neurophysiol Suppl 52:97–103. 10590980

[B59] Sakai K, Ugawa Y, Terao Y, Hanajima R, Furubayashi T, Kanazawa I (1997) Preferential activation of different I waves by transcranial magnetic stimulation with a figure-of-eight-shaped coil. Exp Brain Res 113:24–32. 10.1007/BF02454139 9028772

[B60] Sangari S, Perez MA (2019) Imbalanced corticospinal and reticulospinal contributions to spasticity in humans with spinal cord injury. J Neurosci 39:7872–7881. 10.1523/JNEUROSCI.1106-19.2019 31413076 PMC6774405

[B61] Sangari S, Perez MA (2020) Distinct corticospinal and reticulospinal contributions to voluntary control of elbow flexor and extensor muscles in humans with tetraplegia. J Neurosci 40:8831–8841. 10.1523/JNEUROSCI.1107-20.2020 32883710 PMC7659455

[B62] Schaefer DM, Flanders A, Northrup BE, Doan HT, Osterholm JL (1989) Magnetic resonance imaging of acute cervical spine trauma. Correlation with severity of neurologic injury. Spine (Phila Pa 1976) 14:1090–1095. 10.1097/00007632-198910000-00011 2588058

[B63] Schirmer CM, Shils JL, Arle JE, Cosgrove GR, Dempsey PK, Tarlov E, Kim S, Martin CJ, Feltz C, Moul M, Magge S (2011) Heuristic map of myotomal innervation in humans using direct intraoperative nerve root stimulation. J Neurosurg Spine 15:64–70. 10.3171/2011.2.SPINE1068 21476796

[B64] Smith V, Maslovat D, Carlsen AN (2019) StartReact effects are dependent on engagement of startle reflex circuits: support for a subcortically mediated initiation pathway. J Neurophysiol 122:2541–2547. 10.1152/jn.00505.2019 31642402 PMC6966318

[B65] Steriade M, Pare D, Parent A, Smith Y (1988) Projections of cholinergic and non-cholinergic neurons of the brainstem core to relay and associational thalamic nuclei in the cat and macaque monkey. Neuroscience 25:47–67. 10.1016/0306-4522(88)90006-1 3393286

[B66] Tapia JA, Tohyama T, Poll A, Baker SN (2022) The existence of the StartReact effect implies reticulospinal, not corticospinal, inputs dominate drive to motoneurons during voluntary movement. J Neurosci 42:7634–7647. 36658461 10.1523/JNEUROSCI.2473-21.2022 PMC9546468

[B67] Tazoe T, Perez MA (2017) Cortical and reticular contributions to human precision and power grip. J Physiol 595:2715–2730. 10.1113/JP273679 27891607 PMC5390869

[B68] The Health and Safety Executive (2005) Control of noise at work regulations. The Stationery Office, London.

[B81] Tokimura H, Ridding MC, Tokimura Y, Amassian VE, Rothwell JC (1996) Short latency facilitation between pairs of threshold magnetic stimuli applied to human motor cortex. Electroencephalogr Clin Neurophysiol 101:263–272. 10.1016/0924-980x(96)95664-7 8761035

[B69] Twitchell TE (1951) The restoration of motor function following hemiplegia in man. Brain 74:443–480. 10.1093/brain/74.4.443 14895765

[B70] Uchino Y, Sato H, Sasaki M, Imagawa M, Ikegami H, Isu N, Graf W (1997) Sacculocollic reflex arcs in cats. J Neurophysiol 77:3003–3012. 10.1152/jn.1997.77.6.3003 9212252

[B71] Valls-Solé J, Rothwell JC, Goulart F, Cossu G, Muñoz E (1999) Patterned ballistic movements triggered by a startle in healthy humans. J Physiol 516:931–938. 10.1111/j.1469-7793.1999.0931u.x 10200438 PMC2269293

[B72] Vavrek R, Pearse DD, Fouad K (2007) Neuronal populations capable of regeneration following a combined treatment in rats with spinal cord transection. J Neurotrauma 24:1667–1673. 10.1089/neu.2007.0290 17970629

[B73] Wakabayashi Y, Komori H, Kawa-Uchi T, Mochida K, Takahashi M, Qi M, Otake K, Shinomiya K (2001) Functional recovery and regeneration of descending tracts in rats after spinal cord transection in infancy. Spine (Phila Pa 1976) 26:1215–1222. 10.1097/00007632-200106010-00009 11389386

[B74] Watson SR, Colebatch JG (1998a) Vestibulocollic reflexes evoked by short-duration galvanic stimulation in man. J Physiol 513:587–597. 10.1111/j.1469-7793.1998.587bb.x 9807006 PMC2231297

[B75] Watson SR, Colebatch JG (1998b) Vestibular-evoked electromyographic responses in soleus: a comparison between click and galvanic stimulation. Exp Brain Res 119:504–510. 10.1007/s002210050366 9588785

[B76] Zaaimi B, Edgley SA, Soteropoulos DS, Baker SN (2012) Changes in descending motor pathway connectivity after corticospinal tract lesion in macaque monkey. Brain 135:2277–2289. 10.1093/brain/aws115 22581799 PMC3381720

[B77] Zaaimi B, Soteropoulos DS, Fisher KM, Riddle CN, Baker SN (2018) Classification of neurons in the primate reticular formation and changes after recovery from pyramidal tract lesion. J Neurosci 38:6190–6206. 10.1523/JNEUROSCI.3371-17.2018 29793974 PMC6031583

[B78] Ziemann U, Rothwell JC (2000) I-waves in motor cortex. J Clin Neurophysiol 17:397–405. 10.1097/00004691-200007000-00005 11012042

[B80] Ziemann U, Tergau F, Wassermann EM, Wischer S, Hildebrandt J, Paulus W (1998) Demonstration of facilitatory I wave interaction in the human motor cortex by paired transcranial magnetic stimulation. J Physiol 511(Pt 1):181–190. 10.1111/j.1469-7793.1998.181bi.x 9679173 PMC2231091

[B79] Zörner B, Bachmann LC, Filli L, Kapitza S, Gullo M, Bolliger M, Starkey ML, Rothlisberger M, Gonzenbach RR, Schwab ME (2014) Chasing central nervous system plasticity: the brainstem’s contribution to locomotor recovery in rats with spinal cord injury. Brain 137:1716–1732. 10.1093/brain/awu078 24736305

